# Communicating the Risk of Death from Novel Coronavirus Disease (COVID-19)

**DOI:** 10.3390/jcm9020580

**Published:** 2020-02-21

**Authors:** Tetsuro Kobayashi, Sung-mok Jung, Natalie M. Linton, Ryo Kinoshita, Katsuma Hayashi, Takeshi Miyama, Asami Anzai, Yichi Yang, Baoyin Yuan, Andrei R. Akhmetzhanov, Ayako Suzuki, Hiroshi Nishiura

**Affiliations:** 1 Graduate School of Medicine, Hokkaido University, Kita 15 Jo Nishi 7 Chome, Kita-ku, Sapporo-shi, Hokkaido 060-8638, Japan; tootsieroll2910@gmail.com (T.K.); seductmd@med.hokudai.ac.jp (S.-m.J.); natalie.linton@med.hokudai.ac.jp (N.M.L.); ryokinoshita@med.hokudai.ac.jp (R.K.); katsuma_hayashi@eis.hokudai.ac.jp (K.H.); aanzai@eis.hokudai.ac.jp (A.A.); lukeyang1993@eis.hokudai.ac.jp (Y.Y.); baoyinyuan@outlook.com (B.Y.); akhmetzhanov@med.hokudai.ac.jp (A.R.A.); akmskorokoro@gmail.com (A.S.); 2 Osaka Institute of Public Health, Nakamichi 1-3-69, Higashinari, Osaka 537-0025, Japan; takeshi.j.miyama@gmail.com; 3 Core Research for Evolutional Science and Technology (CREST), Japan Science and Technology Agency, Honcho 4-1-8, Kawaguchi, Saitama 332-0012, Japan

**Keywords:** fatality, virulence, virus, statistical estimation, emerging infectious diseases

## Abstract

To understand the severity of infection for a given disease, it is common epidemiological practice to estimate the case fatality risk, defined as the risk of death among cases. However, there are three technical obstacles that should be addressed to appropriately measure this risk. First, division of the cumulative number of deaths by that of cases tends to underestimate the actual risk because deaths that will occur have not yet observed, and so the delay in time from illness onset to death must be addressed. Second, the observed dataset of reported cases represents only a proportion of all infected individuals and there can be a substantial number of asymptomatic and mildly infected individuals who are never diagnosed. Third, ascertainment bias and risk of death among all those infected would be smaller when estimated using shorter virus detection windows and less sensitive diagnostic laboratory tests. In the ongoing COVID-19 epidemic, health authorities must cope with the uncertainty in the risk of death from COVID-19, and high-risk individuals should be identified using approaches that can address the abovementioned three problems. Although COVID-19 involves mostly mild infections among the majority of the general population, the risk of death among young adults is higher than that of seasonal influenza, and elderly with underlying comorbidities require additional care.

## 1. Introduction

The novel coronavirus disease that appeared in late 2019 (COVID-19) has spread to the majority of East and Southeast Asian countries, and has resulted in a substantial number of deaths [1]. To understand the severity of infection, i.e., the virulence of the causative agent of COVID-19, the common epidemiological practice is to estimate the case fatality risk (CFR) as the risk of death among cases (for the sake of practical interpretation, we refer to it as the case fatality risk rather than the case fatality rate [2]). Depending on the CFR value, the government response toward COVID-19 may vary, and estimates of CFR can also influence the strictness of policy judgement and the extent of containment and mitigation measures. For instance, a well-known CFR estimate for severe acute respiratory syndrome (SARS) in Hong Kong in 2003 was approximately 17% [3], roughly indicating that one out of five diagnosed cases would die of the disease. SARS containment measures were implemented as early as possible due to high estimates of CFR. The total volume of deaths, i.e., mortality, is determined by the product of the CFR and the total number of cases; it should be remembered that our perceived severity of the COVID-19 epidemic can be directly influenced by the absolute number of deaths.

It has been less than two months since the emergence of COVID-19 gained international recognition, and during this early phase the statistical estimation of the CFR is complicated by a number of technical obstacles. It is necessary to (i) account for the time delay from illness onset to death, (ii) define the population considered in the CFR denominator (how we define a case), and (iii) quantify the heterogeneity in the risk of death. Each of these requires a sophisticated modeling approach and detailed case dataset in addition to a simple division of the number of deaths by the number of cases. In the ongoing epidemic of COVID-19, all these aspects have not yet been fully addressed, although the global public health community is obliged to continually confront the epidemic and make political decisions encompassing travel restrictions, containment measures, and mitigation strategies. To accomplish the scientific assessment of the severity and sufficiently understand pitfalls surrounding the associated debates, we aim to guide the readers to understand the likely severity of COVID-19 and direct the course of future research.

## 2. Graduating from Simple Division

CFR is often measured by simply dividing the number of deaths from a disease by the number of cases of a disease as a function of time. If $C_{t}$ and $D_{t}$ are the cumulative numbers of confirmed cases and deaths on day *t* of the epidemic, the simple calculation of the CFR among confirmed cases (cCFR), $p_{t}$ on day *t* is:

(1) $$p_{t}=\frac{D_{t}}{C_{t}},$$

which is known to be flawed, frequently underestimating the actual cCFR [4].

The flaw is especially apparent during the early stage of an epidemic when the number of cases is growing exponentially. The denominator in this phase contains many cases who have recently become ill and have not spent a sufficient number of days ill for them to die of COVID-19. Thus, the simplistic division given by (1) typically underestimates the risk of death among confirmed cases though it may also possibly overestimate it if only a small number of cases and deaths are observed.

The need to account for the time required for cases to die is referred to in statistics as a right censoring issue. During the course of updating epidemiological observations at each latest time point, the case count data is right censored, so the use of the following statistical model addressing this feature is usually employed:

(2) $$p_{t}^{\prime }=\frac{D_{t}}{\sum_{s=1}^{t-1}c_{t-s}f_{s}},$$

where $c_{t}$ is the daily number of cases (i.e., incidence) and $f_{s}$ is the probability density function of the time from illness onset to death—the relative frequency of the time from illness onset to death among fatal cases. See Jung et al. [5] as an example of application of (2) for the ongoing COVID-19 epidemic.

Table 1 compares the cCFR (adjusted estimate) and the value resulting from simple division (not adjusting for the time from illness onset to death). The cCFR using simple division (1) for Hubei province was 2.5%, while addressing the delay from illness onset to death by using (2) yielded a cCFR estimate of 18.0% [6,7]. This has also been seen among cases outside mainland China as cCFR has been measured at 0.4% with division by (1), while 1–5% with adjustment (2).

Considering that the estimated mean time from illness onset to death can be as long as 20 days [5], there may be no deaths yet observed in Chinese cities with recent epidemic spread, which leads to underestimation of the risk of death. It must be remembered that the abovementioned issue can be neglected when the epidemic comes close to its end; even the division (1) converges to the unbiased value of the cCFR. However, during the course of the epidemic, especially in its early phase, the division-based value (1) can increase as a function of time, and such an increase must not be interpreted as a signature of evolution of the virulence (which was partly the case during SARS outbreak, 2003). Even with the adjustment, the 95% confidence intervals may be very wide due to a small number of cases. We therefore advise that estimation of the cCFR must be updated over time in order to reduce the uncertainty that stems from the sampling error [6,7].

## 3. Infection Fatality Risk (IFR): All Infected Individuals as the Denominator

In addition to the issue of statistical right censoring, we have another epidemiological issue of observation, which is referred to as ascertainment bias. When a patient contracts mild illness, the patient may not seek medical attendance. Even with an outpatient visit, physicians may not suspect COVID-19 (there are many reports of cases seeking healthcare multiple times after illness onset and before being recognized and isolated as a suspect case in the current epidemic), because they are unable to distinguish it from other viral respiratory infections based on clinical signs and symptoms alone. Then, the denominator population (the confirmed cases $C_{t}$ in (1)) represents only the tip of the iceberg in terms of the true number of infections, and thus the risk of death among confirmed cases is overestimated. 

Nishiura et al. estimated the ascertainment bias using data on Japanese evacuees from Wuhan [8]. From the end of January 2020, the Japanese government offered chartered flights for a total of 565 Japanese residents living in Wuhan as an exceptional evacuation procedure. All evacuees were interviewed by physicians upon arrival and were admitted to hospitals if they had symptoms that required hospitalization, while the remaining evacuees were quarantined at a hotel or dormitory for 14 days. Those who tested positive from reverse transcriptase polymerase chain reaction (RT-PCR) were isolated at designated hospitals. Eight evacuees had positive RT-PCR results during or shortly after entry screening, while 67 had at least one symptom suggestive of upper respiratory tract viral infection. When the size of the infected evacuees was eight cases, the estimated ascertainment rate for Wuhan was as low as 9.2% [8]. In other words, only 9.2% of infected individuals were estimated to be confirmed in Wuhan, whereas the actual number of infections can be 1/0.092 ≈ 11-fold more than the current number of confirmed infections. What the estimate of 9.2% also indicates is that the cCFR value is likely 11 times greater than the risk of death among all infected individuals, which is referred to as the infection fatality risk (IFR) [9]. For instance, if the value of cCFR is 5–8% as estimated earlier in [5], the IFR can be in a range of 0.5–0.7% out of all infected individuals. This value is also in line with another estimate of the IFR at 0.8% (95% CI: 0.4, 3.0) [6]. When the risk of death appears to be small, the disease can be subjectively perceived as mild for a majority of the general population. However, the risk among elderly with underlying comorbidities may be substantial.

## 4. Additional Calibrations of the Ascertainment Rate

During quarantine, the health of the Japanese evacuees was continuously monitored and RT-PCR testing regularly conducted, and an additional five cases were diagnosed. In total, there were 13 infected evacuees including five asymptomatic individuals as of 16 February 2020. That is, the proportion of infected individuals increased from 8/565 to 13/565—i.e., more than a 50% increase in the prevalence of the infection, and associated lowered ascertainment rate of less than 9.2%.

As another issue, it should be remembered that the evacuees have been diagnosed by RT-PCR testing method which involves two problems. First, the virus detection window was set to be identical to the mean serial interval at 7.5 days [10], but there has been no strict biological justification that the virus is detectable during this time period. An estimated mean infectious period of 3.6 days was also used [11], but there is no direct theoretical link between infectious period and virus positive period. Second, the sensitivity of RT-PCR testing is known to be limited. In fact, the sensitivity to correctly diagnose SARS by RT-PCR test ranged from 44% to 80% [12]. It was found that the sensitivity was greatly influenced by viral shedding and was dependent on the date of sample collection with respect to illness onset [13]. These factors also reduce the previously mentioned ascertainment rate for COVID-19 and indicate that the estimate is likely less than 9.2%. Therefore, the actual number of total cases infected with SARS-CoV-2 causing COVID-19 is likely to be more than 11-fold the number of observed cases as of 16 February 2020.

Let *N* and *C*(*t*) represent the population size of Wuhan City and the cumulative number of cases, respectively. Supposing that the fraction ascertained was *q*, the balance equation for the risk of infection reads:

(3) $$\frac{13}{565s}=\frac{C\left(t\right)T}{qN},$$

where *s* and *T* are the sensitivity and the detection window of RT-PCR testing, respectively. Varying *s* and *T* in the range of plausible values (e.g., *s* to be 30%, 50%, and 80% and *T* to be 1, 3, and 5 days), the ascertainment rate *q* can be estimated from a binomially distributed likelihood function as shown elsewhere [8].

While the ascertainment rate was originally estimated at 9.2%, the plausible value could be smaller if we shorten the virus detection window and account for the limited sensitivity of RT-PCR while updating the number of infected individuals to be 13/565 (Table 2). For instance, the point estimate of the ascertainment rate in Wuhan ranges from 0.2% to 0.6%, if we assume that the virus detection window is 1 day only. This would yield an IFR range of 0.02–0.05%. When we set the detection window at 5 days, the range of ascertainment rate on the other hand becomes as high as 1.1% to 3.1%, and the IFR is on the order of 0.10% to 0.27%. It could still be the case that the IFR varies depending on the actual sensitivity and virus detection time windows, and influences our subjective judgement regarding the severity of COVID-19 infection.

In addition, it must be noted that deaths may also be underascertained [9]. If this is believed to be the case, death counts need to be estimated (e.g., by measuring the excess mortality), and real time modeling becomes even more challenging.

## 5. Heterogeneous risk of Death

The IFR for the entire population is greatly influenced by the composition of the cases with respect to age and underlying comorbidities [14]. Hence, it is more insightful in practice to clarify this subject and to identify individuals who are at high risk of death. To do so, a non-parametric survival analysis could be applied as it was done during the SARS outbreak [15]. Moreover, a mixture of logistic model with survival analysis could help identify the risk factors even with small sample sizes of patients, as was done for Middle East respiratory syndrome (MERS) [16]. To follow a similar approach, we let *b*_i_ be the risk of death of an individual *i*, and write the logit model in the form

(4) $$\ln\left(\frac{b_{i}}{1-b_{i}}\right)=a_{0}+\overset{N}{\underset{k=1}{\sum }}a_{k}x_{k,i},$$

where $a_{0}$ is an intercept of linear predictor, $a_{k}$ is the coefficient of variable *k*, and $x_{k,i}$ is the *k*-th variable of individual *i*. *N* is the total number of explanatory variables. The likelihood function to parameterize the linear predictor is

(5) $$L\left(a;\alpha ,\beta ,t_{m}\right)=\underset{i\in A}{\prod }S\left(t_{m}-\alpha_{i};b_{i}\right)\underset{i\in B}{\prod }b_{i}f\left(\beta_{i}-\alpha_{i}\right),$$

where $\alpha_{i}$ and $\beta_{i}$ are the observed dates of symptom onset and death of case *i*, respectively, and *A* and *B* represent groups of cases who have survived and died by the most recent calendar time $t_{m}$. From (5), parameters that govern the coefficients $a_{k}$ are estimated, allowing for prediction of risk of death depending on age and underlying comorbidities.

Using (5) with application to MERS, the risk of death among patients older than 60 years of age was estimated to be 9.3 times greater than those in the younger age group [16]. Likewise, the CFR for SARS was estimated to be 43.3% in those aged 60 years or older and 13.2% among those younger than 60 years of age [15]. As age and underlying comorbidities of the deceased cases start to become evident [17], we may be able to offer a tailor-made prediction of the risk of death among high risk populations.

## 6. Conclusions

Finally, we discuss an additional aspect of validation. In Japan, there was a large cluster of confirmed cases (*n* = 218 as of 14 February 2020) who acquired infection of COVID-19 on a cruise ship (all passengers were tested for infection using RT-PCR). Most passengers are 60 years of age or older—a group which frequently have comorbidities. Out of 218 cases, there are 10 critically ill patients as of 15 February 2020. Assuming that one out of five critically ill patients dies of infection, the risk of death among these high-risk individuals is approximately 1%. Considering that many patients have just developed illness and will manifest complications in a matter of 7–10 days [18], there could be additional critically ill patients in the future. Thus, from a matter of plausibility, it is likely that the IFR among elderly with underlying comorbidities is 1% or greater. Considering this, and also the population-average in Table 1, the risk of death among young adults would be smaller than that of older adults, e.g., at most 0.1%–0.2%. Whether to perceive this estimate as severe or moderate is a matter for discussion, but we consider 0.1%–0.2% among young adults to be unusually high. At the very least, this article indicates that the severity of COVID-19 is less than SARS and greater than that of seasonal influenza.

In conclusion, this article reviewed several key epidemiological problems for assessment of the severity of COVID-19. First, division of the cumulative number of deaths by that of cases should be adjusted by the delay in time from illness onset to reporting. Second, assessing only confirmed cases among all infections can offer limited insights into the severity among all infected individuals. Third, ascertainment bias and IFR are reduced by a shorter virus detection window and lower RT-PCR sensitivity. In the ongoing COVID-19 epidemic, health officials will need to confront the uncertainty in the risk of death, and prompt identification of high-risk individuals using the approaches covered in this article is needed. Subjectively, COVID-19 involves mild infections among the general population, but the risk of death among young adults is higher than for seasonal influenza, and the mortality among older adults with comorbidities requires close attention.

## Figures and Tables

**Table 1:** Confirmed case fatality risk (cCFR) with and without adjustment of the time from illness onset to death.

Locations	Unadjusted cCFR as of 14 February 2020 [1]	Adjusted cCFR [6]	95% Confidence Intervals of cCFR [6]
Hubei	2.5%	18%	11–81%
Outside mainland China	0.4%	1–5%	1–85%

**Table 2:** Ascertainment rate and infection fatality risk estimates estimated from Japanese evacuees from Wuhan City, China.

Sensitivity	Virus Detection Window (Days)					
	1 Day		3 Days		5 Days	
	*q* (%)	IFR (%)	*q* (%)	IFR (%)	*q* (%)	IFR (%)
30%	0.2 (0.2, 0.3)	0.02 (0.02, 0.02)	0.7 (0.6, 0.8)	0.06 (0.05, 0.07)	1.1 (0.9, 1.4)	0.10 (0.08, 0.12)
50%	0.4 (0.3, 0.5)	0.03 (0.03, 0.04)	1.1 (0.9, 1.5)	0.10 (0.08, 0.13)	1.9 (1.5, 2.5)	0.16 (0.13, 0.22)
80%	0.6 (0.4, 0.9)	0.05 (0.04, 0.08)	1.8 (1.3, 2.6)	0.16 (0.12, 0.23)	3.1 (2.2, 4.4)	0.27 (0.19, 0.38)

95% confidence intervals, derived from profile likelihood, are shown in parenthesis.
